# A Post-Treatment Method to Enhance the Property of Aerosol Jet Printed Electric Circuit on 3D Printed Substrate

**DOI:** 10.3390/ma13245602

**Published:** 2020-12-08

**Authors:** Bing Wang, Haining Zhang, Joon Phil Choi, Seung Ki Moon, Byunghoon Lee, Jamyeong Koo

**Affiliations:** 1 School of Mechanical and Aerospace Engineering, Nanyang Technological University, Singapore 639798, Singapore; wangbing@e.ntu.edu.sg (B.W.); m160034@e.ntu.edu.sg (H.Z.); joonphil.choi@ntu.edu.sg (J.P.C.); 2 Strong-Field and Ultrafast Photonics Lab, Key Laboratory of Trans-Scale Laser Manufacturing Technology, Ministry of Education, Faculty of Materials and Manufacturing, Beijing University of Technology, Beijing 100124, China; 3 Beijing Engineering Research Center of Laser Technology, Beijing University of Technology, Beijing 100124, China; 4 Singapore Centre for 3D Printing, School of Mechanical and Aerospace Engineering, Nanyang Technological University, Singapore 639798, Singapore; 5 Global Technology Center, Samsung Electronics Co., Ltd., Suwon 16677, Korea; bh13.lee@samsung.com (B.L.); jamyeong.koo@samsung.com (J.K.)

**Keywords:** 3D printing, anti-high temperature, microelectronic device, sensor

## Abstract

Aerosol jet printing of electronic devices is increasingly attracting interest in recent years. However, low capability and high resistance are still limitations of the printed electronic devices. In this paper, we introduce a novel post-treatment method to achieve a high-performance electric circuit. The electric circuit was printed with aerosol jet printing method on an ULTEM substrate. The ULTEM substrate was fabricated by the Fused Deposition Modelling method. After post-treatment, the electrical resistance of the printed electric circuit was changed from 236 mΩ to 47 mΩ and the electric property was enhanced. It was found that the reduction of electric resistance was caused by surface property changes. Different surface analysis methods including scanning electron microscopy (SEM) and x-ray photoelectron spectroscopy (XPS) were used to understand the effectiveness of the proposed method. The results showed that the microsurface structure remained the same original structure before and after treatment. It was found that the surface carbon concentration was significantly increased after treatment. Detailed analysis showed that the C-C bond increased obviously after treatment. The change of electrical resistance was found to be limited to the material’s surface. After polishing, the circuit resistance was changed back to its original value. As the electric circuit is the basic element of electric devices, the proposed method enables the fabrication of high performance devices such as capacitors, strain gauge, and other sensors, which has potential applications in many areas such as industrial, aerospace, and military usage.

## 1. Introduction

Direct writing is a widely used 3D printing method for electronic device fabrication due to its low-cost, high customization, and flexibility [1,2,3]. 3D printing or additive manufacturing is an advanced manufacturing technology developed in recent years, which is widely used in aerospace, defense, and other industries [4,5,6]. Materials are joined layer by layer through the process. Compared with the traditional manufacturing methods, direct writing is a simple and fast process of creating high precision features and customized components. There are various direct writing methods such as inkjet printing [3], screen printing [7] and aerosol jet printing [2]. Recently, there is an increasing number of research studies related to electric devices printed by direct writing technologies, such as the direct printing of supercapacitors [8], transistor circuits [9,10], solar cells [7,11], and organic light-emitting devices (OLEDs) [12].

Aerosol jet printing is a relatively new developed direct writing method [13,14,15]. It is a contactless printing method with high resolution, high flexibility, and enables printing on various substrates such as metals, semiconductors, and polymers. It was initially developed for the 3D printing of electronic circuitry and the printing resolution is typically up to 10 µm [16,17]. During the printing process, metal ink/electronic ink is accurately and precisely deposited onto the substrates through aerodynamic focusing [18]. Materials able to suspend in aerosol are compatible with this technique, besides metal inks, ceramics, polymers, carbon nanotubes, and biomaterials are also able to be deposited [17]. In general, it is most widely used for electronic circuit or device printing. Clifford et al. investigated the aerosol jet printed humidity sensor on pre-packaged integrated circuits. It shows a good response to humidity in the range of 40% to 80% [19]. Cantu et al. investigated the aerosol jet printed electrochemical sensors for protein detection. It shows the aerosol jet printed sensors can detect lower current density and has a lower deviation compared with screen-printed sensors [20]. Cao et al. reported that the aerosol jet printed carbon nanotube thin film transistor. The printed transistor shows high reliability and good performance up to 1000 cycles of bending test [21]. Maiwald et al. studied the functionality of strain gauge sensors fabricated using aerosol jet printing [22]. Zhao et al. reported the direct strain gauge printing by aerosol jet method on composite structures [23]. Rahman et al. investigated the aerosol jet printed strain sensors at high temperature up to 500 °C, and the reliability and performance of the sensors were tested [24]. The quality of the aerosol jet printed device is of great importance in applications. However, in the current status, the low capability and high resistance are still limitations of the aerosol jet printed electronic devices [25]. Fundamental research works have investigated the quality of aerosol jet printed electric circuits [17,26,27]. Bourassa et al. reported a water vapor assisted sintering process. With the presence of 10 g water in oven during the sintering process, the resistivity of the aerosol jet printed circuit was found to be significantly reduced. It was shown that the reduction of resistivity was due to the larger connected area formed during the sintering process [28]. Efimov et al. investigated the effect of substrate temperature on the aerosol jet printing sintering process. To improve the conductivity, an optimized temperature of 200 °C was obtained [29]. Halonen et al. [30] optimized the sintering temperature to minimize the resistance. It was found that a strong endothermic reaction appeared at temperature range of 160 °C to 210 °C, which indicated sintering via diffusion and partial melting without mass reduction. Hwang et al. [31] reported that the sintering temperature and time affected neck and pore growth which resulted in the specific resistance; the printed Ag nanoparticle ink after sintering at 250 °C for 50 min showed minimum specific resistance. Dana et al. proposed a novel intense pulsed light (IPL) sintering process for aerosol jet printed electric circuit. It was reported that the fresh printed layer could be solidified in milliseconds with IPL post-treatment, which has an advantage for polymer substrate printing [32]. Mahajan et al. studied the effect of the ratio of sheath gas flow rate to carrier gas flow rate (focusing ratio) on the printed quality. It was found that the line width decreased the focusing ratio, while line thickness increased when increasing the focusing ratio [26]. An analytical study was conducted by Binder et al. to identify the effect of process parameters and predict the printed line width [27]. Smith et al. investigated the effect of gas flow rate, focus ratio, and substrate temperature on aerosol jet printed line quality [17]. Zhang et al. proposed a hybrid machine learning method to optimize the printing parameter and determine the aerosol jet printing operation window [18]. Goh et al. studied the adhesiveness, conductivity, and wetting property of aerosol jet printed circuit. It was found that the adhesion could be increased with a plasma treatment before printing [33]. There is an increasing number of research studies focusing on the quality improvement of the printed circuit. However, most of the research is limited to optimization in processing parameters and the high resistance of the aerosol jet printed circuit is still not properly solved.

Compared with traditional fabricated silicon-based electronic devices, electronic devices fabricated on polymer substrates can maintain its electrical continuity in both the original state and under deformation. ULTEM is a high strength plastic with high temperature resistance, high chemical resistance, high dielectric strength, and extremely strong and stiff. It is widely used for electrical connectors, manifolds, medical components, and scientific parts. It has become a popular material for FDM 3D printing due to its stability and adhesive property [34,35,36]. ULTEM 9085 is a new commercial 3D printing material that can be used for aerospace applications [37].

In this paper, we introduce a novel post-treatment method for printing a high electrical performance circuit. Firstly, the electric circuit is printed by aerosol jet printing method on a FDM fabricated ULTEM 9085 substrate with optimized printing parameters. Then, the sample is treated for a high temperature and high humidity post process. After the treatment, the resistance of the electric circuit can be reduced significantly. SEM investigation is conducted for surface morphology analysis. Surface chemical analysis is performed with XPS analysis.

## 2. Materials and Methods

**Figure 1:**
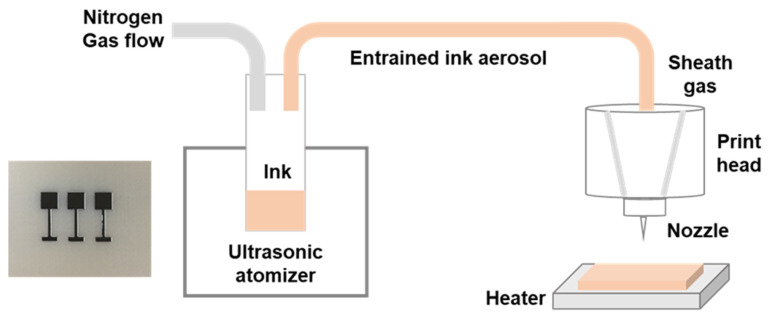
Illustration of the aerosol jet printing process and an image of the printed electronic circuit.

A 40 mm × 20 mm × 5 mm ULTEM 9085 substrate was fabricated by a Fortus 450 mc machine from Stratasys (Eden Prairie, MN, USA). The machine build envelop was 406 × 355 × 406 mm. The nozzle size was T16. Air gap and raster angle were 0 mm and +45°/−45°, respectively. The layer thickness was 0.254 mm with a contour width of 0.508 mm. An Aerosol Jet 3D printer from Optomec^®^ (Albuquerque, NM, USA) was employed to conduct the experiment. During the process, firstly the silver ink was atomized by the ultrasonic atomizer, then it was entrained in nitrogen gas carriers. The entrained ink aerosol was then delivered to the print head. In the print head, the ink aerosol was shaped and accelerated by sheath gas to the print nozzle. The aerosol droplet was deposited into the substrate and heated for solvent evaporation as presented in Figure 1. Silver nanoparticle ink (Clariant) diluted with DI water at a ratio of 1:1 was used for the experiment. The atomizer current was 0.3 mA. The nozzle tip height was 3 mm with a diameter of 150 μm. Printing speed was set to be 1 mm/s and the ink temperature was fixed at 20 °C. The flow rate of carrier gas and sheath gas were set to 30 and 45 sccm (standard cubic centimeters per minute), respectively. The printed line width was 40 µm with an overlap of 25%. The printed layer was fixed at 3. The thickness of each printed layer is measured to be around 3 µm. After printing, the sample was sintered for 2 h at 200 °C. Aerosol jet printing is a complicated process and main process parameters affect the printing quality significantly. In this paper, all the process parameters were optimized with a hybrid method. Firstly, Latin hypercube sampling was applied to design the experiment, and then the printing quality was analyzed quantitatively with respect to the process parameters. After that, the optimal operating process window of aerosol jet printing was identified by a support vector machine approach. A detailed information of the optimization process can be found in Zhang, et al. [18].

The printed sample was then subjected to two types of post-processes: (1) Treatment A: a cycled treatment at −40 °C to 85 °C for 100 rounds and (2) Treatment B: high temperature treatment at 80 °C with high humidity 80% for 120 h as shown in Table 1. The conductivity before and after treatment was measured. The surface structure and surface chemistry were investigated with SEM (5600LV, Jeol Asia Pte Ltd., Tokyo, Japan) and XPS (VG ESCALAB 220i-XL, Thermo Scientific, East Grinstead, West Sussex, UK). 

## 3. Results and Discussion

**Figure 2:**
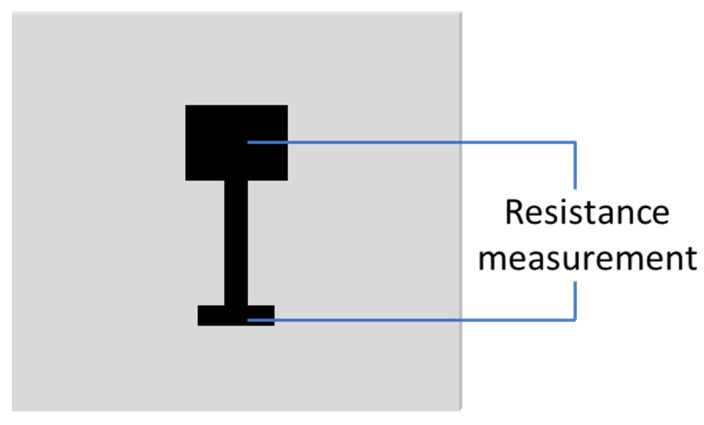
Demonstration of resistance measurement.

The 3D printed circuit is shown in Figure 1. The resistance of the original printed circuit from head to bottom was measured to be 236 mΩ, as demonstrated in Figure 2. The circuit resistance after treatment is shown in Table 2. As depicted in Table 2, after Treatment A (100 cycles post-treatment at temperature varied from −40 °C to 85 °C), the resistance changed to 230 mΩ. After Treatment B (120 h post-treatment at 80 °C with 80% humidity environment), the resistance reduced to 47 mΩ.

**Figure 3:**
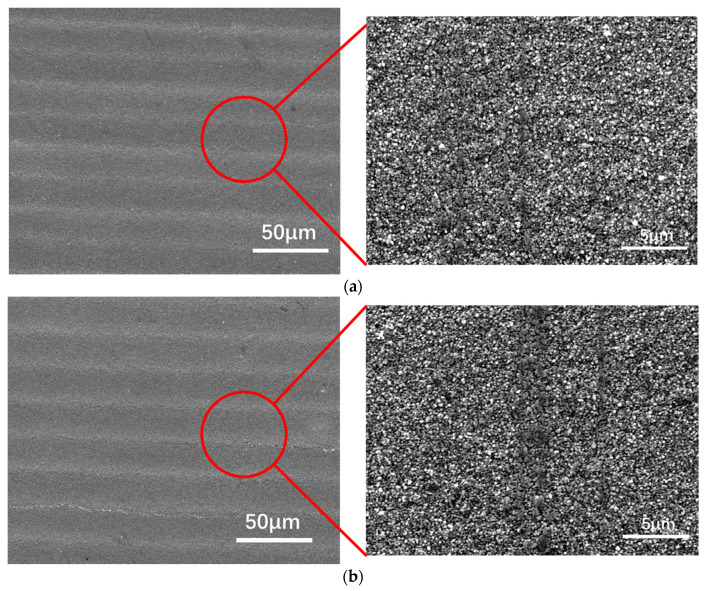
SEM image of (**a**) original sample and (**b**) sample after treatment at 80 ℃ and 80% humidity for 120 h.

From Table 2, the reduction of resistance is significantly after treatment B. For aerosol jet printed electric circuits, the sample is normally considered as 2D structures with a few micrometers in thickness [39]. The reduction can be caused by the changes of surface property. To further investigate this phenomenon, surface properties including both surface chemistry and surface structure were examined before and after the treatment. The surface structure was investigated with SEM as shown in Figure 3. Figure 3a shows the SEM image of the original electric circuit surface, we can see the aerosol jet printed lines clearly along the ULTEM substrate. At a high magnification of 5000×, the silver nanoparticles can be seen Figure 3a, right side. The surface structure after post-treatment at 80 °C with high humidity 80% for 120 h is shown in Figure 3b. The aerosol jet printed lines were still clear and at high magnification, and the nanoparticles were observed. From this figure, it is clear that there were no obvious microstructure changes found on the surface before and after treatment.

**Figure 4:**
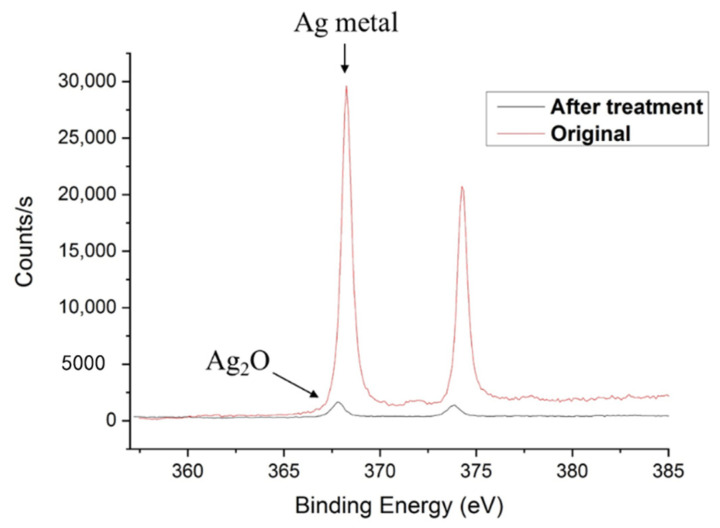
XPS analysis of silver element before and after treatment B, detailed data results are shown in Table 2 with relative errors.

XPS analysis was conducted for surface chemistry investigation. Both wide scans of all the chemical elements and a narrow scan of the focused elements were performed. As shown in Table 3, there are four elements detected on the original surface: oxygen, silver, carbon, and chlorine. The original oxygen atomic concentration was measured to be 18.3% and after treatment, it increased to 23.4%. The increase of oxygen indicates the oxidation on the surface. The original atomic concentration of silver was 38.9% and after treatment, the silver concentration reduced to 1.0%. The decrease of silver concentration and increase of oxygen was possibly caused by the oxidation of silver. A detailed XPS analysis of silver element is shown in Figure 4. We can see the silver peak of original sample was around 368.2 eV, which is the peak of Ag metal. While after treatment, the silver peak shifted to 367.9 eV, which is the peak of Ag_2_O. The detailed XPS analysis of silver element proved that the Ag metal was oxidized into Ag_2_O after the post-treatment. The high temperature and humidity environment may increase the oxidation speed of silver. Under a high humidity environment, a large amount of sliver cation is generated. The generated Ag^+^ was reacted with hydroxide anion (OH^−^) in water, thus silver oxide was generated on the surface. The present of high temperature also rapid up the reaction speed. On the other hand, there are no obvious changes in the surface property of the sample after one month under room temperature and normal humidity. There are few amounts of chlorine detected on the original sample surface, and it was disappeared after treatment. These small amounts of chlorine may from the add-in material of the original silver dilute. After treatment, the sample surface was oxidized, and the carbon concentration increased significantly. The absorbed oxygen and carbon atom from the ambient environment may cover the small amount of chlorine elements, thus the chlorine elements are not detected after treatment. The hypothesis can be further proved by that after polishing, the chlorine elements were detected again.

**Figure 5:**
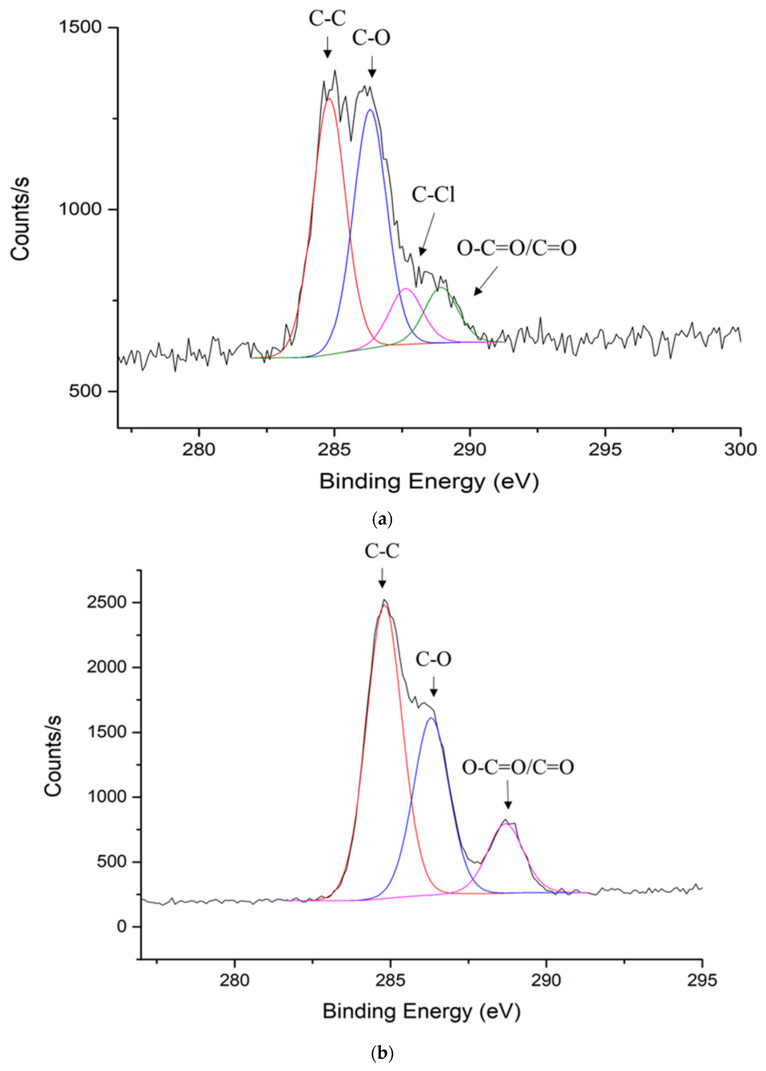
Carbon analysis of (**a**) Original sample; (**b**) After treatment B.

Beside silver element, carbon element concentration was also changed significantly. From Table 3, the carbon atomic concentration increased from 41.2% to 75.6% after the post-treatment. Detailed XPS analysis for carbon element was conducted as shown in Table 4 and Figure 5a,b. As shown in Table 4, there are four peaks detected on the original sample: C-C bond, C-O bond, C-Cl bond, and O-C=O/C=O bond. The C-C bond atomic concentration increased from 42.3% to 54.4% after treatment. The C-O bond decreased from 39.4% to 32. 8%. The O-C=O/C=O bond increased from 9.2% to 12.8%. The C-Cl bond was not detected after treatment. Since the total carbon concentration was increased significantly from 41.2% to 75.6%, even though the bond atomic concentration decreased. Overall, they all increased compared with the original sample.

From the literature, when silver elements exposed to the atmosphere environment for a long term, oxidation and carbon contamination could be significant [40,41]. The amount of silver decreased significantly due to the increase of oxygen and carbon species on the sample surface. The increased carbon concentration and associated nonpolar carbon group formation decreased the surface free energy [42,43,44,45,46]. Generally, the absorption of carbon item from the environment occurs in the long term [47,48], while the annealing process can accelerate the process in a short time [45,48,49]. There are several explanations for the increase of carbon concentration in the atmosphere including: the airborne hydrocarbon contamination from the atmosphere [45,46]; the absorption of other organic matters such as PDMS contamination from furnace [49], or other organic contamination like carboxylate from ambient air; the decomposition of carbon dioxide into carbon [43] with active magnetite; the partial deoxidation of the oxidized metal [50,51]. Further research has shown that the presence of common atmosphere gases such as CO_2_, O_2,_ and N_2_ is not the main reason causing the carbon concentration changes, instead, the absorption of organic compounds seems to have a more significant influence on the carbon concentration [52,53]. These further supported with other research works showing that immersion or store in organic rich media will accelerate the carbon absorption process as in [44,54,55,56]. It seems the organic contamination during the annealing process could be the most possible reason. The mechanism of resistance reduction is still not clear. As reported in [28], the formation of larger connected silver ink areas during the sintering process with water could be a possible reason. The removal of organic capping agent at low temperature from the contacting gap could result in the resistivity reduction. On the other hand, it was reported by Matikainen et al. [40] that the carbon contamination from reaction with hydrocarbons in the atmosphere could result in the formation of thin carbon layers on the surface. Also, a graphene carbon layer was reported to form on metal surfaces under high temperature treatment with the presence of carbon sources [57,58]. Either hydrocarbon gases or solid polymer films can be the source for graphene layer generation on metal surfaces [57,58].

In this case, the microscale SEM image was taken and showed no obvious changes. However, nanoscale changes of silver ink were not examined. The connected area in high a humidity environment may increase as reported in [28]. On the other hand, the increase of C-C bond on the surface could be the main reason causing the resistance reduction. A carbon layer was generated during the process thus enhanced the sample conductivity significantly (resistance dropped from 236 mΩ to 47 mΩ). Besides the small amount of hydrocarbon gases in the air, ULTEM (polyetherimide), which contains a great amount of carbon atoms, can act as carbon sources as well. Since the printed ULTEM substrate has a porous structure, there are quite a number of un-melted and deposited powders attached to the substrate. Under high humidity and high temperature environment, the powder can be dissolved into the atmosphere. The pyrolysis occurred on the surface as time going on. In addition, the process can be catalyzed with the presence of silver nanoparticles [58]. On the other hand, the formed carbon layer can act as a protection to avoid further oxidization on the electric circuit surface.

**Figure 6:**
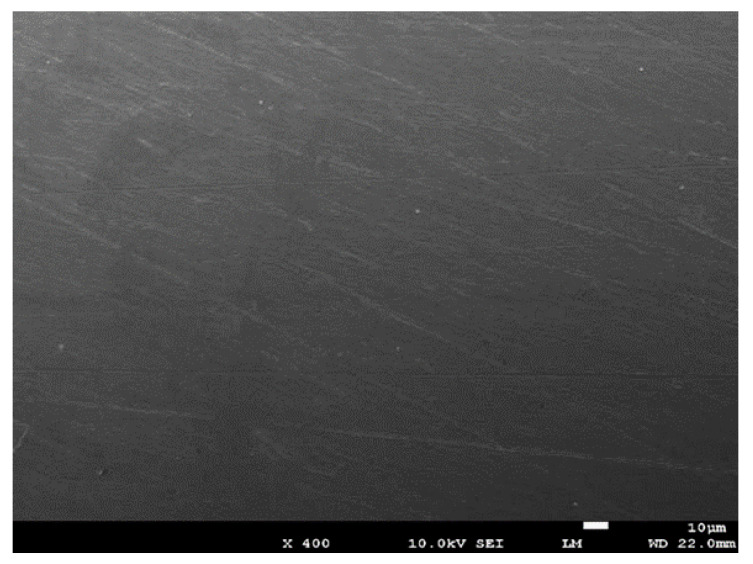
SEM image of the polished sample.

To investigate the reduction of resistance on the material surface, the printed samples was polished with a 1200 grade sandpaper for 2 s to remove the outer surface. The SEM image of a polished sample is shown in Figure 6. After polishing, the printed lines disappeared, and the sample became flat. The resistance of the polished sample was measured to be 242 mΩ, which was near to the original sample resistance. This indicates the resistance reduction after treatment was mainly attributed to the thin layer at the surface of the printed sample. Table 5 shows the XPS analysis of the polished sample. After polishing, the carbon element concentration reduced to 46.1% and the atomic concentration of silver increased back to 39.5%, which was quite near to the original value. The resistance recovered back after polishing could be due to the removal of the carbon layer on the surface.

## 4. Conclusions

In this paper, we introduced a novel post-treatment method to increase the performance of the aerosol jet printed electric circuit. Original electric resistance was measured to be 236 mΩ, after 100 cycles treatment with temperature varied from −45 °C to 85 °C, the resistance changed a bit to 230 mΩ. Meanwhile, after treatment at 80 °C with 80% humidity for 120 h, the resistance was successfully reduced to 47 mΩ. SEM examination showed the surface microstructure remained as the same original structure before and after treatment. XPS analysis showed the silver metal was oxidized after treatment and the carbon element was significantly increased, particularly in the C-C bond. The oxidation of silver surface was due to the presence of a high humidity environment, while the carbon element increase was most probably due to the organic contamination in the atmosphere during the treatment process. A carbon layer was formed on the sample surface, thus resulting in the reduction of electrical resistance. For further investigation, the top few layers of the treated sample were removed by a polishing method. After polishing, the sample resistance increased back to 242 mΩ, which was near to its original resistance. XPS measurement showed that after the polishing, the surface chemistry changed near to the original surface. The carbon layer formed on surface was removed, thus the sample’s conductivity returned to its origin. The findings and proposed treatment method have potential usage as carbon protection coating of electronic devices and enable the printing of high-performance electronic devices.

Overall, the current investigation is a preliminary research that is limited to the discussion of the effect of the surface carbon layer and the related electrical circuit conductivity. To be specific, the proposed post-treatment approach would be beneficial to achieve the high electrical performance while minimizing the electrical resistance. For future work, efforts are required to study the influences of sintering parameters, i.e., sintering temperature and time, on the defect, microstructure, and conductivity of printed sensors.

## Figures and Tables

**Table 1:** Sample post-treatment methods.

Sample	Post-Treatment Method
Original	NA (Stored in air)
Treatment A	−40 °C to 85 °C for 100 cycles
Treatment B	80 °C, 80% humidity for 120 h

**Table 2:** Measured sample resistance before and after treatment.

Sample	Resistance (mΩ)
Original	236 ± 1.8
Treatment A	230 ± 1.8
Treatment B	47 ± 6.9
Silver film [38]	45

**Table 3:** Atomic concentration of original sample and sample after treatment B.

Atomic Concentration (%)		
Sample	Original	After treatment B
O 1s	18.3 ± 4.7	23.4 ± 2.7
Ag 3d	38.9 ± 1.8	1.0 ± 6.7
C 1s	41.2 ± 2.3	75.6 ± 0.8
Cl 2p	1.6 ± 18.5	-

**Table 4:** XPS analysis of carbon element.

Atomic Concentration (%)		
Sample	Original	After treatment B
C 1s 284.8 (C-C)	42.3 ± 3.1	54.4 ± 1.2
C 1s 286.3 (C-O)	39.4 ± 3.1	32.8 ± 1.2
C 1s 287.6 (C-Cl)	9.2 ± 2.9	-
C 1s 288.6(O-C=O/C=O)	9.2 ± 2.8	12.8 ± 1.0

**Table 5:** XPS analysis of polished sample.

Atomic Concentration (%)			
O 1s	Ag 3d	C 1s	Cl 2p
12.0 ± 13.1	39.5 ± 2.1	46.1 ± 1.9	2.4 ± 8.2
